# Pomegranate Extract Potentiates the Anti-Demineralizing, Anti-Biofilm, and Anti-Inflammatory Actions of Non-Alcoholic Mouthwash When Associated with Sodium-Fluoride Trimetaphosphate

**DOI:** 10.3390/antibiotics11111477

**Published:** 2022-10-25

**Authors:** Gabriela Lopes Fernandes, Ana Paula Miranda Vieira, Marcelle Danelon, Nayara Gonçalves Emerenciano, Andresa Aparecida Berretta, Andrei Felipe Moreira Buszinski, Juliana Issa Hori, Mikhael Haruo Fernandes de Lima, Thaila Fernanda dos Reis, Jessica Aparecida de Lima, Alberto Carlos Botazzo Delbem, Sónia Carina Morais da Silva, Debora Barros Barbosa

**Affiliations:** 1 Graduate Program of Dental Science, School of Dentistry, Araçatuba, São Paulo State University (UNESP), Araçatuba 16015-050, São Paulo, Brazil; 2 School of Dentistry, University of Ribeirão Preto—UNAERP, Ribeirão Preto 14096-039, São Paulo, Brazil; 3 Department of Restorative Dentistry, School of Dentistry, Araçatuba, São Paulo State University (UNESP), Araçatuba 16015-050, São Paulo, Brazil; 4 Apis Flora Industrial and Comercial Ltd. Ribeirão Preto 14020-670, São Paulo, Brazil; 5 Department of Biochemistry and Immunology, University of São Paulo Ribeirão Preto, Ribeirão Preto 14049-900, São Paulo, Brazil; 6 Department of Dental Materials and Prosthodontics, School of Dentistry, Araçatuba, São Paulo State University (UNESP), Araçatuba 16015-050, São Paulo, Brazil; 7 National Institute for Agrarian and Veterinary Research (INIAV), Vairão, 4485-655 Vila do Conde, Portugal

**Keywords:** *Punica granatum*, polyphosphates, fluoride, dental enamel, antimicrobial, anti-inflammatory

## Abstract

This study investigated the anti-caries and anti-inflammatory effects of mouthwash formulations containing *Punica granatum* (pomegranate) peel extract (PPE), sodium-trimetaphosphate, and low concentrations of fluoride. PPE was characterized using high-performance liquid chromatography (ellagic acid and punicalagin). Total phenolics were quantified among formulations, and their stability was analyzed for 28 days. The formulation effects were evaluated as follows: (1) inorganic component concentration and reduced demineralization on bovine enamel blocks subjected to pH cycling; (2) anti-biofilm effect on dual-biofilms of *Streptococcus mutans* ATCC 25175 and *Candida albicans* ATCC 10231 treated for 1 and 10 min, respectively; and (3) cytotoxicity and production of inflammatory mediators (interleukin-6 and tumor necrosis factor-alpha). The formulation containing 3% PPE, 0.3% sodium-trimetaphosphate, and 225 ppm of fluoride resulted in a 34.5% surface hardness loss; a 13% (treated for 1 min) and 36% (treated for 10 min) biofilm reduction in *S. mutans*; a 26% (1 min) and 36% (10 min) biofilm reduction in *C. albicans*; absence of cytotoxicity; and anti-inflammatory activity confirmed by decreased interleukin-6 production in mouse macrophages. Thus, our results provide a promising prospect for the development of an alcohol-free commercial dental product with the health benefits of *P. granatum* that have been recognized for a millennium.

## 1. Introduction

Dental caries is the most common polymicrobial oral disease worldwide [1], and, although knowledge of caries has increased, researchers and dentists still struggle to find better alternatives for the prevention and treatment of this disease [2]. The mouth contains polymicrobial flora composed of bacteria and yeast that play important roles in fermentation of sugars in acids, leading to enamel demineralization. The main microorganisms involved in this process are *Streptococcus* spp., *Staphylococcus* spp., *Lactobacillus* spp., and *Candida* spp. [3,4,5,6].

The primary modes of preventing the development and progression of dental caries are brushing, flossing, and mouthwash use [7,8,9]. Mouthwashes have advantages, such as easy application and antimicrobial and anti-inflammatory action, are more available in less accessible regions than brushes and floss, and are also a topical source of fluoride (F) [7,10]. Previous studies have proved that addition of sodium-trimetaphosphate (TMP) can optimize the ability of F to reduce enamel demineralization, and that this action may be related to the ability of this phosphate to bind the dental surface, thereby changing its permeability to calcium (Ca) and F ions [10,11,12,13,14,15].

In this sense, nature provides a plant source with enormous medicinal properties, *Punica granatum*. Considered “a pharmacy unto itself,” this fruit features important properties, such as antimicrobial, anti-inflammatory, astringent, and antioxidant properties, and has no significant toxicity [16,17,18]. *P. granatum* has been proven beneficial for reducing recurrent aphthous stomatitis pain and complete healing time [19,20] as a preventive and therapeutic aid in periodontal disease [21,22,23] and an antifungal agent against *Candida* spp. [24,25]. Plants may utilize multiple strategies to deal with microorganisms that have evolved over time [26]. Therefore, their secondary metabolites represent a large library of compounds that may potentiate the effects of known antibiotics and be important sources of new drugs or compounds suitable for further modification [27]. The main chemical compounds present in *P. garantum* are eicosanoic, linolenic conjugated, linolenic alpha, oleic, palmitic, punicic, stearic, citric, and malic acids; phenolic compounds, such as gallic acid, coumaric acid, catechin, phloridzin, quercetin, and protocatechuic, chlorogenic, cafeic, and ferulic acids [28,29]. According to Al-obaidi et al., ellagic acid, punicalagin, punicic acid, flavonoids, anthocyanidins, anthocyanins, estrogenic flavanols, and flavones have greater therapeutic activity [30].

Although the antimicrobial effects of *P. granatum* are well described in the literature, its action on the demineralization dental process when associated with TMP and F is unknown. Considering the health risks associated with the prolonged use of chemicals in conventional mouthwashes, the benefits of bioactive compounds present in plants for preventing oral and dental diseases, as well as the remarkably increased interest in herbal medicine, we aimed to determine the anti-caries and anti-inflammatory effects of non-alcoholic mouthwashes produced with *P. granatum* (pomegranate) peel extract (PPE), TMP, and low concentrations of F.

## 2. Results

### 2.1. Phytochemical profile of PPE

**Figure 1:**
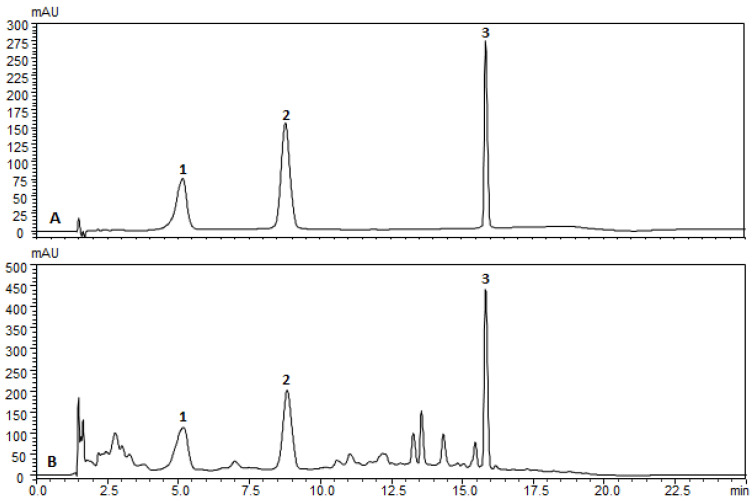
Punicalagin α (1), Punicalagin β (2), and Ellagic acid (3); (**A**) reference standards and (**B**) pomegranate peel extract fingerprint obtained by high-performance liquid chromatography according to the conditions presented in the methodology.

PPE samples were extracted in methanol, and the chemical profiles were defined using chromatography and compared with commercial chemical standards. The retention times of the punicalagin isomers (α and β) and ellagic acid were analyzed in PPE (glycolic extract). PPE presented similar retention times compared with the reference standards (Figure 1). The spectral profiles of the samples were similar, and the peak purity was found to be close to 1.0000. The concentrations of pomegranate biomarkers in PPE were: punicalagin α = 2.14 ± 0.006 mg g^−1^, punicalagin β = 2.286 ± 0.03 mg g^−1^, and ellagic acid = 0.458 ± 0.006 mg g^−1^.

Folin–Denis colorimetric assay revealed that PPE is rich in phenolic compounds. Table 1 illustrates the mean of the total phenolics expressed as gallic acid (mg mg^−1^) found in each formulation containing 3% PPE, as well as in E (formulation containing PPE), ETF1 (3% PPE + 0.2% TMP + 100 ppm F), and ETF2 (3% PPE + 0.3% TMP + 225 ppm F) formulations. The total phenolic content (mg g^−1^) in each formulation containing PPE was similar (average 11.54 mg g^−1^), corresponding to 10% of the pomegranate peel extract.

### 2.2. Anti-Demineralization Effect

The average pH of all mouthwash solutions was adjusted to 7.0. The mean surface hardness of all blocks was 364.6 (standard deviation 9.8; Knoop hardness (KHN); (*p* = 0.533)). No significant differences were observed among groups after random allocation (*p* = 0.474). The use of formulation F2 (225 ppm of F) resulted in approximately 12% surface hardness loss (%SH) compared with that of F1 (100 ppm of F). The demineralization of the enamel surface was less in samples treated with the ETF2 formulation, resulting in approximately 37%, 47%, and 49% SH loss compared with that of F2, TF2, and CM (essential oil commercial mouthwash), respectively (*p* < 0.001). In addition, the capacity to reduce the subsurface hardness integrated loss (ΔKHN) was higher (approximately 29%) with ETF2 (*p* > 0.001) than with TF2 (*p* < 0.001), followed by ETF1 = F2 = TF1 > CM > E = W (deionized water) (*p* < 0.001) (Table 2).

Similar and higher amounts of F were observed with formulations F2, TF2, ETF2, and CM (*p* > 0.001). With F2, enamel Ca concentration increased by approximately 40% compared with that with F1 (*p* < 0.001). The highest Ca concentration was observed with ETF2: when compared to CM, there was an increase of 70% (*p* < 0.001). The phosphorus (P) concentrations were similar for all formulations, except forW, E, and F1, which showed the lowest values (*p* < 0.001) (Table 2).

### 2.3. Formulation Stability—F Quantification and Determination of Minimal Inhibitory Concentration

The amount of F^-^ in formulations containing sodium-fluoride (TF2 and ETF2) remained stable (225 ± 20 ppm). As expected, the N and TF2 formulations showed no minimal inhibitory concentration. E and ETF2 maintained the same minimal inhibitory concentration ranges of 4.022.01 mg mL^−1^ for *S. mutans* and 1.05–0.5025 mg mL^−1^ for *C. albicans* throughout the study period (0, 7, 14, 21, and 28 days).

### 2.4. Anti-Biofilm Effect

PPE associated with TMP and F in mouthwash formulations reduced viable *C. albicans* and *S. mutans* cells in biofilms.

In vitro biofilm assays were performed using important oral pathogens involved in dental caries as reference strains to verify whether PPE could inhibit *C. albicans* and *S. mutans* proliferation in association with TMP and F. Thus, the biofilms were grown for 24 h on hydroxyapatite (HA) discs and then exposed to the mouthwash formulations for 1 min and 10 min. In addition, to elucidate the mouthwash effect in the biofilm environment, we measured the pH of 24-h biofilms treated for 1 min and 10 min.

**Figure 2:**
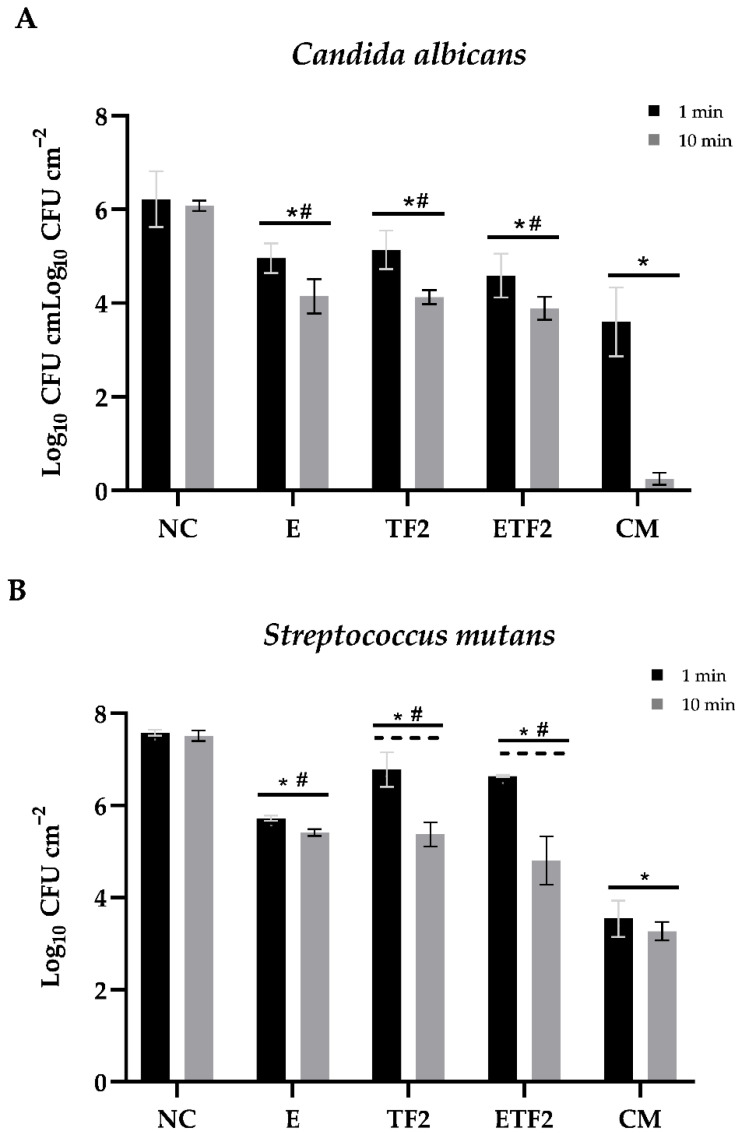
NC: negative control; E: formulation with 3% pomegranate peel extract; TF2: formulation with 0.3% sodium-trimetaphosphate + 225 ppm of fluoride; ETF2: formulation with 3% pomegranate peel extract + 0.3% sodium-trimetaphosphate + 225 ppm of fluoride; CM: essential oil commercial mouthwash. Means and standard deviations of viable (Log_10_ cm^2^) *Candida albicans* ATCC 10231 (**A**) and *Streptococcus mutans* ATCC 25175 cells (**B**), and biofilms after treatment with each formulation for 1 min and 10 min. (**A**): Asterisk indicates *p* < 0.05 versus negative NC; # indicates *p* < 0.05 of ETF2 (after 1 min), TF2 (after 1 min), and E (after 1 min) versus CM (after 1 min) (one-way ANOVA followed by Holm–Sidak test). (**B**): Asterisk indicates *p* < 0.05 versus NC; # indicates *p* < 0.05 of ETF2, TF2, and E versus CM; ---- indicates *p* < 0.05 of ETF2 (after 1 min) versus ETF2 (after 10 min), and TF2 (after 1 min) versus TF2 (after 10 min) (one-way ANOVA followed Holm–Sidak post hoc test).

As shown in Figure 2, all mouthwash formulations significantly reduced the number of biofilm cells for both *C. albicans* and *S. mutans*. Similarly, the positive control (CM), formulations E, TF2, and ETF2 produced a significant reduction in *C. albicans* biofilm cells compared with that in the negative control group (*p* < 0.001). Although there was no significant difference between 1 min and 10 min of treatment with the E, TF2, and ETF2 formulations, the percentage of *C. albicans* biofilm reduction increased considerably after 10 min of exposure (Table 3). These results also demonstrate that *S. mutans* biofilm cells treated for 10 min with the ETF2 and TF2 formulations were significantly more susceptible than those treated for 1 min (*p* < 0.001), whereas, for formulation E and the positive control, treatment time had no significant influence on the reduction in *S. mutans* cells (*p* > 0.05). Among the ETF2, TF2, and E mouthwash formulations, the highest rates of viable cell reduction were exhibited by the ETF2 formulation after 10 min of treatment regardless of the microorganism tested (Table 3). As shown in Table 4, the lowest pH was found in biofilms treated with the TF2 formulation regardless of exposure time.

### 2.5. Anti-Inflammatory Effect

**Figure 3:**
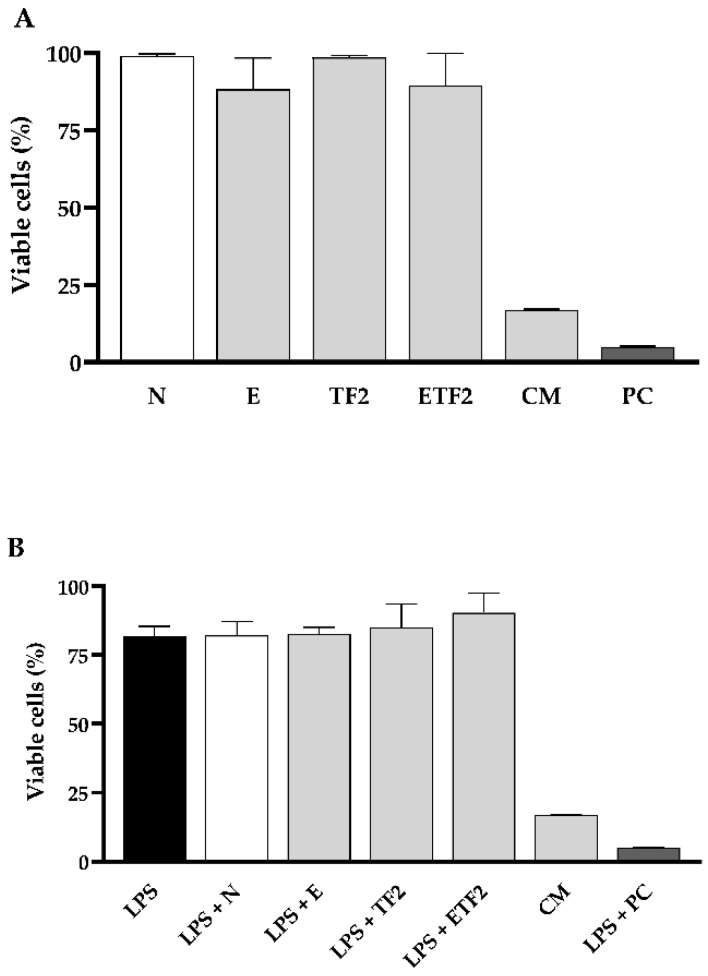
Percentage of cell viability of bone-marrow-derived macrophages (BMDMs) by MTT assay after treatment with each formulation. (**A**) BMDMs were not previously stimulated and (**B**) BMDMs were stimulated with lipopolysaccharide (LPS, 1 μg mL^−1^) for 4 h and then exposed to N: formulation without fluoride (F), sodium trimetaphosphate (TMP) and pomegranate peel extract (PPE); E: formulation with 3% PPE; TF2: formulation with 0.3% TMP + 225 ppmF; ETF2: formulation with 3% PPE + 0.3% TMP + 225 ppmF; CM: essential oil commercial mouthwash; PC (positive control, triton) for 2 h.

**Figure 4:**
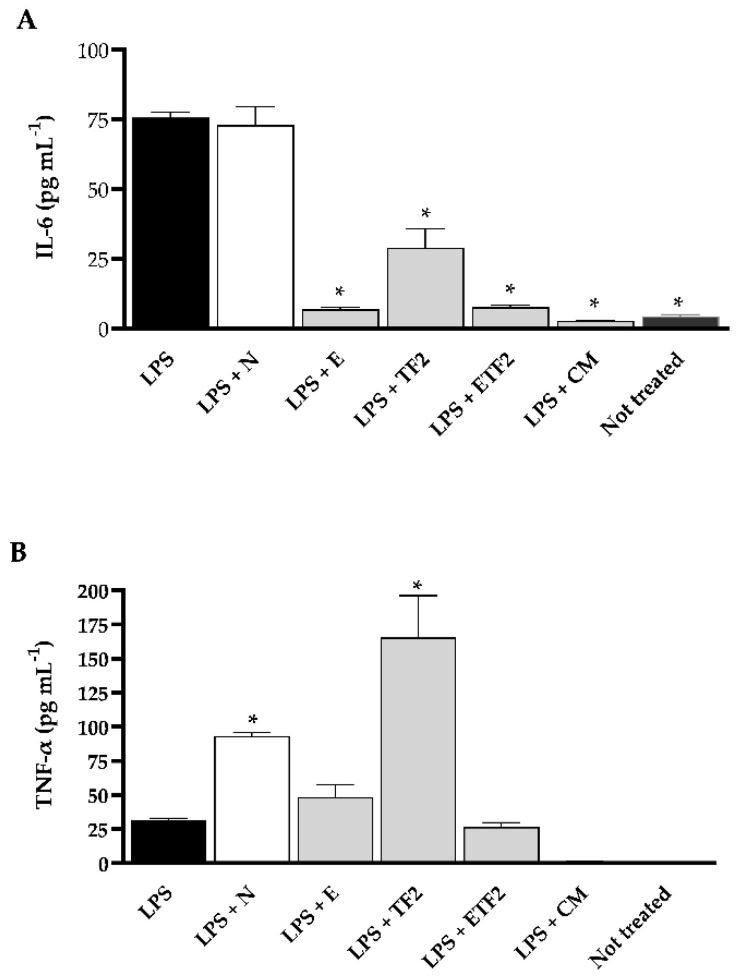
Evaluation of pro-inflammatory cytokine production by bone-marrow-derived macrophages (BMDMs). Expression of interleukin-6 (IL-6) lipopolysaccharide (LPS)-induced (**A**) and tumor necrosis factor-alpha (TNF-α) LPS-induced (**B**) production in BMDMs supernatants culture after treatment with N: formulation without fluoride (F), sodium trimetaphosphate (TMP) and pomegranate peel extract (PPE); E: formulation with 3% PPE; TF2: formulation with 0.3% TMP + 225 ppm F; ETF2: formulation with 3% PPE + 0.3% TMP + 225 ppm F; CM: essential oil commercial mouthwash for 2 h. “Not treated” denotes cells that were not stimulated with LPS. (**A**) IL-6 and (**B**) TNF-α concentrations in culture supernatants were measured in triplicate using an ELISA assay. Data were pooled from representative of three independent experiments (mean ± SD). Asterisks indicate statistical significance regarding cells stimulated only with LPS, (**A**) *p* ≤ 0.001 and (**B**) *p* ≤ 0.05 (one-way ANOVA followed by the Tukey multiple comparisons test).

In order to verify if the formulations also presented an anti-inflammatory effect, we conducted an experiment using murine bone-marrow-derived macrophages (BMDMs) treated or not with lipopolysaccharide (LPS) from *Escherichia. coli*, which is already known to stimulate inflammatory cytokines, and checked if any formulation would be able to decrease the production of the pro-inflammatory cytokines interleukin-6 (IL-6) and tumor necrosis factor-alpha (TNF-α). The treatment with CM was expressively cytotoxic, showing only ~17% of the viability of BMDMs for both assays with (Figure 3A) and without (Figure 3B) previous LPS stimulation. Consequently, there was no IL-6 and TNF-α production by cells treated with this product (Figure 4). Conversely, formulations E, TF2, and ETF2 did not cause any cytotoxic effects (Figure 3) and showed an immunomodulatory effect for IL-6 in LPS pre-treated cells (Figure 4A). Besides, TF2 stimulated the highest production of TNF-α (Figure 4B).

## 3. Discussion

Here, PPE was characterized in relation to dry matter, total phenolics, ellagic acid, and punicalagin content. The extract contained high levels of polyphenols, especially ellagitannins (ellagic acid and punicalagin), which are bioactive compounds responsible for many pharmacological properties [31]. Concerning the total phenolic content, phenolic components were only detected in the formulations containing PPE (E, ETF1, and ETF2).

Regarding the potential of enamel demineralization reduction with the proposed formulations, the main finding of this study was that the association of PPE (3%) with TMP (0.3%) and F (225 ppm) (ETF2) presented the best results for reduction in mineral loss from the enamel surface and subsurface and was more effective than formulations F2, TF2, and ETF1. Although the formulation containing extract (E) alone did not show a statistically significant difference from the W) in all analyses performed (Table 2), the association of PPE with TMP and F improved the reduction in mineral loss from the enamel. CM had similar results to formulations F2 (225 ppm) and F1 (100 ppm) in reducing mineral loss from the enamel surface and subsurface, respectively, which might be due to the presence of F at 220 ppm in CM. Other ingredients present in CM, such as eucalyptol, thymol, and methyl salicylate, do not have any antidemineralizing action: Zero et al. demonstrated that an essential-oil-based mouthwash (without F) did not promote effective enamel remineralization in an in situ caries model [32].

The actions of TMP and F in the anti-caries process have already been well established [10,33,34,35]. Studies have shown that TMP can be adsorbed on the enamel surface, thereby reducing demineralization because this process can hinder acid diffusion and alter the affinity between enamel and salivary proteins [36,37,38]. TMP adsorbs into the enamel, forming a reticular layer that covers the enamel surface and decreases Ca phosphate apatite precipitation within the enamel pores. Therefore, phosphate diffusion is facilitated, enabling Ca and F ions to lead to less demineralization in the external and internal parts of the lesion (Table 2).

However, the association of pomegranate with TMP and F has not yet been investigated. Although no studies in the literature have evaluated the anti-demineralization action of *P. granatum*, this effect has been proven in other plants, such as *Galla chinesis*, *Acacia*, *Salvadora persica*, and *Camellia sinensis* [39,40]. The literature clearly shows that *P. granatum* is rich in polyphenols (such as punicalin, punicalagin, and gallic and ellagic acid) [29], as well as Ca, magnesium, P, potassium, and sodium, mostly found in the peel of this fruit [41]. Zhang et al. evaluated the action of *Galla chinensis* extract on bovine enamel matrix subjected to acidic challenges and found that monomeric and polymeric polyphenols interacted with this organic enamel matrix (through covalent, ionic, and hydrogen bonding or hydrophobic processes), leading to a metamorphism of this matrix that precipitated and decreased ion loss in the enamel structure [42,43]. Another possible action is the binding of compounds present in the extract to the crystal surface of enamel, thus preventing its demineralization, in addition to facilitating deposition of more ions on the surface (through ion carriers) [44,45]. Other studies have shown that gallic acid (present in *P. granatum*) can function as a Ca ion transporter, favoring the remineralization process [46]. Thus, this external source of Ca increases the availability of these ions to the TMP-F layer and diffusion into the lesion (Table 2) and reduces demineralization in the deeper part of the subsurface lesion. In addition, a more pronounced reduction in the subsurface lesion (ΔKHN) suggests that, under clinical conditions, a subsurface lesion takes more time to develop when compared with conventional treatments (i.e., F alone). These results can be considered significant from a clinical point of view, especially when the lesion may take longer to develop and is associated with other preventive interventions. Therefore, the mechanism described above explains the superior effects of ETF2. Since the TMP does not benefit the precipitation of CaF_2_ in the enamel [47,48], this effect should reduce the obstruction of the pores of the enamel surface facilitating the diffusion of ions in the enamel. The association of the active agents 3% PPE + 0.3% TMP + 225 ppm F led to better results in reducing demineralization on the surface (%SH), in depth (ΔKHN), in addition to the higher formation of Ca ions in the enamel, which leads to the hypothesis that TMP binds to Ca^2+^, CaF^+^, and PPE ions, forming a complex with greater potential in reducing caries lesions, providing an additional benefit.

Although the beneficial effects of *P. granatum* in the process of tooth enamel demineralization and remineralization have not been evaluated in previous studies, many studies have proven its anti-inflammatory, antioxidant, and antimicrobial activities [18,49], which are compatible with our results. The antimicrobial effect of the dual-biofilm formulations of *C. albicans* and *S. mutans* formed after 24 h and treated for 1 min and 10 min showed that, although there was no statistically significant difference between the ETF2, TF2, and E formulations, they all significantly reduced the number of viable cells. Moreover, formulation ETF2 was superior to the others, except for reduction in *S. mutans* after 1 min of treatment. The mechanism of action of the compounds present in the formulations and the specific characteristics of the microorganisms tested (bacteria and fungi) may have directly influenced these results. In a study by de Oliveira et al., *P. granatum* extract promoted *S. mutans* inhibition, whereas *C. albicans* showed low sensitivity [50].

*S. mutans* is a facultative anaerobic Gram-positive bacterium present in dental biofilms and is one of the numerous etiological factors for dental caries [51]. The virulence factors of this microorganism are acidogenic (mainly production of lactic acid) and aciduric properties. In addition, *S. mutans* uses sucrose from the diet to synthesize large amounts of extracellular polysaccharides, mostly glucans, synthesized by glucosyltransferases [38,51,52]. *C. albicans*, in contrast, is an opportunistic pathogenic fungus [51,53], and its virulence is related to the transition from its yeast form to hyphae [54]. This fungus is known to colonize caries lesions [52] and has the ability to produce acids and extracellular polysaccharides [51]. The symbiotic relationship between *S. mutans* and *C. albicans* contributes to increased biofilm virulence [55].

The phenolic compounds present in the extracts are responsible for their antimicrobial activity [56]. In general, the antimicrobial activity of PPE may be due to the presence of substantial quantities of phytocompounds, such as flavonoids (quercetin, rutin, naringenin, luteolin, pelargonidin, prodelphinidin, and kaempferol) and hydrolyzable tannins (including methyl gallate, peduncalagin, punicalin, punicalagin, gallic, and ellagic acid) [57]. These phytocompounds act on cell walls and membranes of microorganisms by precipitating proteins, in addition to inhibiting enzymes, such as glycosyltransferase, making it difficult to adhere to microorganisms [58].

Scalbert showed that tannins have antimicrobial activity against fungi and bacteria [59]. Koo et al. observed that cranberry juice inhibited the activity of glycosyltransferase in *S. mutans* biofilms [60]. Brighenti et al. observed through proteomic studies that *Psidium cattleianum* extract inhibited proteins responsible for RNA synthesis, and, in *S. mutans,* the reduction in seven important proteins in carbohydrate metabolism and lactic acid production was observed, which is directly linked to development of dental caries [56].

Vasconcelos et al., who tested a gel formulated with *Punica granatum* in *Candida albicans* and *Streptococcus mutans* (isolated and combined), observed significant action against *Candida albicans*, although not as much as the commercial antifungal miconazole [61]. Endo et al. also demonstrated the strong activity of *Punica granatum* crude extract against *C. albicans*, showing morphological changes in the cells through transmission electron microscopy, such as irregular budding patterns and pseudohyphae, thickening of the cell wall, changes in the space between the cell wall and plasma membrane, as well as a reduction in cytoplasmic content [62]. Gulube and Patel showed that *P. granatum* extract affected acid and extracellular polysaccharides production in a biofilm of *S. mutans*, which did not harm the oral microbiota balance [63].

The association of TMP and F with the extract may have contributed to its antimicrobial action. Cavazzana et al. showed that TMP and F reduced the number of viable *S. mutans* cells, and TMP (0.25% without F) decreased the total biomass and extracellular matrix components of *C. albicans* and *S. mutans* biofilms [38]. Another interesting finding in our study is that the biofilm pH after treatment for 1 min and 10 min was acidic, mainly for biofilms treated with formulations ETF2 and TF2, and, even with these values, we observed reductions between 1 and 2 logs for the *C. albicans* and *S. mutans* biofilms when treated with the developed formulations. These pH values were also reported by other authors when biofilms were treated with extracts and F [64,65].

Surprisingly, although CM tested is without alcohol and it has shown the highest antimicrobial capacity among the tested groups, this mouthwash produced the highest cytotoxicity against BMDMs. These results corroborate those of previous studies on the cytotoxicity of CM, which demonstrated its toxicity in human keratinocyte epithelial [66] and mouse calvarial preosteoblast cells [67]. In contrast, our proposed mouthwash formulations without alcohol (N, E, TF2, and ETF2) demonstrated approximately 80% viability of BMDMs cells after 2 h of treatment.

It has already been established that LPS induces the secretion of pro-inflammatory cytokines, such as TNF-α and IL-6, in macrophages. These pro-inflammatory cytokines are multifunctional mediators involved in regulation of immune response and inflammation [68]. Our results showed that the E and ETF2 formulations suppressed IL-6 production in LPS-induced BMDMs. Elevated IL-6 levels are associated with periodontal tissue degradation [69], chronic apical periodontitis [70], and early childhood caries [71]. The IL-6 content in saliva is reportedly higher in patients with periodontitis than in healthy patients [72] and is positively correlated with periodontal lesion severity [73]. In addition, a recent study reported increased IL-6 levels in overweight/obese children with cavitated caries lesion [74].

In contrast, in the BMDMs cells pre-stimulated with LPS, TNF-α pro-inflammatory cytokine production was not reduced, differing from the results previously reported using the *P. granatum* extract [75,76,77]. In our study, pomegranate peel extract was inserted into a formulation with several ingredients. The formulation base (N), without the presence of actives, stimulated the production of TNF-α compared with the formulations containing PPE (E and ETF2). However, these formulations maintained the level of TNF-α compared with that in the group stimulated with LPS; that is, there was no increase in cytokine production. This may be beneficial as TNF-α has an essential physiological role in the immunomodulatory process in infections, and it has been proven that TNF-α-deficient mice had greater susceptibility to infectious agents [78,79].

In conclusion, the addition of PPE (3%) to mouthwash formulations containing TMP (0.3) and F (225 ppm) promoted considerably decreased mineral loss of dental enamel and substantially reduced the cariogenic biofilm formed by *S. mutans* and *C. albicans*. In addition, these formulations were not toxic to BMDMs cells downregulating pro-inflammatory cytokine IL-6. These findings, along with those of further clinical studies, may lead to the development of an alcohol-free commercial dental product with the health benefits of pomegranate that have been recognized for a millennium, and anti-caries properties of TMP and F may become feasible.

## 4. Materials and Methods

### 4.1. Plant Material and Extraction Procedure

*Punica granatum* (pomegranate) peel (dehydrated, crushed, and sterilized) was obtained from a single allotment from the Santos Flora Company (*Santosflora Comércio de Ervas Ltd.*, Mairiporã, SP—Brazil). The product is certified by Food and Drug Administration, in addition to pharmacopeial and microbiological analyses. PPE was obtained by maceration (24 h), followed by a percolation process using ethanol (70° GL) as the extraction solvent until drug exhaustion. The obtained extract was concentrated in a vacuum evaporator until residue (solvent evaporated) and subsequently diluted in propylene glycol to reach 30% *w*/*v* of pomegranate dry matter (measured by evaporation of the solvent in the oven at 100–105 °C until constant weight).

#### Chemical Analysis of PPE by High-Performance Liquid Chromatography

Chromatographic quantification of the pomegranate compounds was performed by high-performance liquid chromatography using a Shimadzu system (Shimadzu Corporation, Kyoto, Japan) consisting of a pump (LC-20AT), diode array detector (SPD-M20A), system controller (CBM-20A), autoinjector (SIL-20A), LC-20AT quaternary pump, and Shimadzu LC solution software. The chromatographic separation and the ellagic acid and punicalagin determination were performed using a reverse-phase Shimadzu Shim-Pack GIST analytical column C18 (100 mm × 4.6 mm × 3 μm) at 30 °C, as described by Santiago et. al, with some modifications [80]. The mobile phase consisted of acetonitrile (phase B) and water containing 5% formic acid (*v*/*v*) (phase A) using the following gradient program: 0–5 min, 97–95% A; 5–10 min, 95–85% A; 10-16 min, 85-70% A; 16–18 min, 70–97% A; 18–25 min, 97% A. The flow rate was 0.8 mL min^−1^. Peaks were determined by comparison with an authenticated ellagic acid (Sigma-Aldrich, St. Louis, MO, USA) and punicalagin α/β (Sigma-Aldrich) standard. PPE samples were diluted in methanol, homogenized in an ultrasonic bath for 30 min, and then filtered (0.45 µL). The injection volume was 5 μL, and we used a wavelength of 260 nm. All samples were prepared in triplicate.

### 4.2. Preparing the Mouthwash Formulations

The formulations were standardized according to their active principle in 3% PPE, 0.2 or 0.3% TMP, and 100 or 225 ppm of F. The formulations also contained stabilizers, microbiological preservatives, chelators, sweeteners, humectants, and water. The pH of all formulations was adjusted to 7.0 (Table 5). The PPE concentration (3%) was determined according to microbiological assays [81] (Supplementary Material—Table S1), and the TMP (0.2 or 0.3) and F (100 or 225 ppm) concentrations were based on previous studies {Favretto, 2013 #523}.

Formulations N (formulation without F/TMP/PPE), E (formulation containing PPE), F1 (100 ppm of F), F2 (225 ppm of F), TF1 (0.2% TMP + 100 ppm of F), TF2 (0.3% TMP + 225 ppm of F), ETF1 (3% PPE + 0.2% TMP + 100 ppm of F), ETF2 (3% PPE + 0.3% TMP + 225 ppm of F), and CM (220 ppm of F, essential oil anticaries alcohol-free commercial mouthwash, Johnson & Johnson© from Brazil—Supplementary Material) were subjected to a pH cycling test. The formulations with the best performance were subsequently tested in dual-biofilm models of *C. albicans* and *S. mutans* reference strains, and their anti-inflammatory capacity was determined.

#### Quantification of Total Phenolics

To verify the total phenolics present in the formulations, an analytical curve of gallic acid was constructed as detailed in a previous study [82,83]. Briefly, the formulations and a standard solution of gallic acid were solubilized in water. The formulations were maintained in an ultrasonic bath for 30 min. A 0.5 mL aliquot was transferred to a 50 mL flask to which 2.5 mL of Folin–Denis reagent (Qhemis-High Purity, Hexis, São Paulo, Brazil) and 5.0 mL of 29% sodium carbonate (Cinética, São Paulo, Brazil) were added. The solutions were incubated in the dark, and the readings were recorded after 30 min using a UVmini-1240 spectrophotometer (Shimadzu Corporation) at 760 nm. All samples were prepared in triplicate.

### 4.3. Experimental Design pH Cycling

Enamel blocks (4 × 4 mm, *n* = 84) of bovine incisors were stored in a 2% formaldehyde solution (pH 7.0) for 30 days at room temperature. The enamel surfaces of the blocks were sequentially polished and selected by an initial surface hardness (SHi, KHN) test of the total blocks and trust interval, and then randomized (SHi: 320.0 to 380.0 KHN) into 7 groups (*n* = 12 per group): formulation N, F1, F2, TF1, TF2, ETF1, and ETF2 (Table 5). The enamel blocks were subjected to pH cycling (demineralization solution (DE) for 6 h (Ca and P 2.0 mmol L^−1^ in acetate buffer 0.075 mol L^−1^, 0.04 μg F/mL at pH 4.7–2.2 mL/mm^2^) and then in a remineralizing solution (RE) for 18 h (Ca 1.5 mmol L^−1^, P 0.9 mmol L^−1^, 0.15 mol L^−1^ KCl in 0.02 mol L^−1^ sodium cacodylate buffer, 0.05 μg F/mL at pH 7.0–1.1 mL/mm^2^) for five days and treated with each formulation twice a day (1 min). Deionized water rinses were performed between each step, and the blocks were placed in a fresh remineralizing solution for the final 2 days [84]. Subsequently, the blocks were assessed for final surface hardness (SHf), %SH, subsurface hardness integrated loss (ΔKHN), and F, Ca, and P concentration in the enamel [35].

#### 4.3.1. Determination of Fluoride in Solutions

The F concentration in the solution was determined using a specific electrode for the F ion (9609 BN; Orion Research Inc., Beverly, MA, USA) attached to an ion analyzer (Orion 720 Aplus; Orion Research Inc.) and calibrated with standards containing 0.25 to 4.00 ppm of F. Primarily, 1.0 mL of each product was dissolved in deionized water and transferred to a volumetric flask. The volume was adjusted to 100 mL using deionized water. Three dilutions were prepared for each product. Subsequently, two 1 mL samples were buffered with total ionic strength adjustment buffer (TISAB II, Sigma aldrich) [85]. The pH of the solutions was determined using a pH electrode (2A09E, Analyzer, São Paulo, Brazil) calibrated with standard pH levels of 7.0 and 4.0 [36].

#### 4.3.2. Experimental Solutions and Treatment with Formulations

The blocks from each group were subjected to five pH cycles at 37 °C during a procedure that lasted for seven days [84]. The blocks were immersed in a demineralizing (DE) solution (2.0 mmol L^−1^ Ca and phosphate in 75 mmol L^−1^ acetate buffer, pH 4.7; 0.04 µg F mL^−1^; 2.2 mL mm^−2^). After 6 h, the blocks were transferred to a remineralizing (RE) solution (RE: 1.5 mmol L^−1^ Ca, 0.9 mmol L^−1^ phosphate, and 150 mmol L^−1^ potassium chloride in 0.1 mol L^−1^ cacodylic buffer, pH 7.0; 0.05 mg F mL^−1^; 1.1 mL mm^−1^) for 18 h. Deionized water rinses were performed between each step. The treatment regimen consisted of a 60-s soak in 1 mL/block of solution under agitation on a rotatory shaker before the solution was changed from DE to RE and from RE to DE (twice a day). Deionized water rinses were performed between each step. In the final two days, the blocks were stored in an RE solution.

#### 4.3.3. Hardness Measurements

The hardness of the enamel surface was determined using a microhardness tester (HMV-2000e, Shimadzu Corporation) and a Knoop diamond under a 25 g-load for 10 s. Five indentations spaced 100 µm apart were made at the center of the SHi. After pH cycling, the SHf was calculated by producing five other indentations (100 μm from the baseline indentations). The %SH was calculated using the following formula: %SH = ([SHf − Shi]/SHi]) × 100. For the analysis of longitudinal hardness, each block was divided in half and one half was embedded in acrylic and polished resin. We used a Micromet 5114 microdurometer (Buehler, Lake Bluff, IL, USA) and Omni Met software (Buehler) with a loading of 5 g for 10 s. A sequence of 14 impressions at distances of 5, 10, 15, 20, 25, 30, 40, 50, 70, 90, 110, 130, 220, and 330 μm from the outer surface of the enamel was made in the central area of the blocks [86]. The integrated hardness area (KHN × μm) from the lesion to the hard enamel was calculated using the trapezoidal rule (GraphPad Prism, version 3.02; GraphPad Software, San Diego, CA, USA) and subtracted from the hard enamel hardness-integrated area to obtain the integrated loss of subsurface hardness (∆KHN).

#### 4.3.4. Analysis of F, Ca, and P Concentration in the Enamel

Blocks (*n* = 12/per group, 2 mm × 2 mm) were obtained from the halves of the original 4 mm × 4 mm specimens that were not embedded and fixed with adhesive glue on a mandrel for straightening. Self-adhesive polishing discs (diameter, 13 mm) and 400-grit silicon carbide (Buehler) were fixed to the bottom of the polystyrene crystal tubes (J-10; Injeplast, Sao Paulo, SP, Brazil). One layer 50.0 ± 0.03 µm thick was removed from each enamel block. After the addition of 0.5 mL hydrogen chloride 1.0 mol L^−1^, it was constantly stirred for 1 h [87], modifying the procedure described by Alves et al. [88]. For the F analysis, a specific electrode 9409BN (Thermo Fisher Scientific, Waltham, MA, USA) and microelectrode reference (Analyser, São Paulo, Brazil) coupled to an ion analyzer (Orion 720A+, Thermo Fisher Scientific) were used. The electrodes were calibrated with standards containing 0.25 to 4.00 μg F mL^−1^ (100 ppm of F, Orion 940907) under the same conditions as the samples. The readings were conducted using 0.25 mL of biopsy solution buffered with the same volume of TISAB II modified with 1.0 mol L^−1^ sodium hydroxide [89]. The results are expressed in μg/mm^3^. Ca analysis was performed using the Arsenazo III colorimetric method [90]. Absorbance readings were recorded at 650 nm using a plate reader (PowerWave 340, Agilent Technologies, Winooski, VT, USA). P was measured as described by Fiske et al., and absorbance readings were recorded at 660 nm [91]. The results are expressed in µg/mm^−3^.

### 4.4. Stability Test of Mouthwash Formulations

ETF2 was selected to be subjected to the stability test based on the National Health Surveillance Agency protocols (Cosmetics Stability Guide, ISBN 85-88233-15-0; Copyright^©^ ANVISA, 2005) and the Guide to Stability Studies (Ordinance No. 593 of 25 August 2000), with controlled temperature and time conditions. TF2, E, and N formulations were also tested as controls. Briefly, samples of each formulation were subjected to alternating cycles of temperature daily, ranging from 40 to −5 °C, for 28 days. After 0, 7, 14, 21, and 28 days, F quantification (described in Section 4.3.1.) and MIC (based on Clinical and Laboratory Standards Institute Documents M27-A2 and M07-A9, with some modifications) against *Candida albicans* American Type Culture Collection (ATCC) 10231 and *Streptococcus mutans* ATCC 25175 were determined.

### 4.5. Anti-Biofilm Activity

#### 4.5.1. Artificial Saliva, Microorganism Strains, and Growth Conditions

The artificial saliva used in this experiment was based on the protocol described by Lamfon et al. [92] (Supplementary Material). Two reference strains from the ATCC were used in this study: *C. albicans* ATCC 10231 and *S. mutans* ATCC 25175. The strains were grown as previously described by Arias et al. *C. albicans* cell quantities were adjusted using a Neubauer chamber and resuspended in saliva at 1 × 10^7^ cells mL^−1^, and *S. mutans* was adjusted by spectroscopy, optical density_640_ nm = 1.6 (EONC Spectrophotometer, Agilent Technologies) at 1 × 10^8^ cells [93].

#### 4.5.2. Biofilm Assay

According to Pandit et al., the HA discs were positioned vertically on supports made of orthodontic wires in 24-well plates (Costar, Corning Inc., Corning, NY, USA) (Supplementary Material) [64]. Before contact with a mixed biofilm of *C. albicans* and *S. mutans*, the discs were conditioned in 2 mL of artificial saliva for 1 h incubated at 37 °C in 5% carbon dioxide (CO_2_)_._ Then, the saliva was removed, and 2 mL of the E, TF2, ETF2 formulations, and CM (positive control) were added and kept in contact with the discs for 1 min. The discs were washed in saline solution, and 2 mL of the dual-inoculum (*C. albicans* and *S. mutans*) was added to the wells containing HA discs that were previously conditioned with each mouthwash. Next, the 24-well plates were incubated (37 °C, 5% CO_2_) for 24 h and treated for 1 min and 10 min with mouthwash.

#### 4.5.3. Number of Cultivable Cells

Colony-forming units (CFUs) from dual-biofilms formed on the HA discs and treated with each formulation were quantified. HA discs were added into Falcon tubes containing 3 mL of saline solution, subjected to an ultrasonic bath (Elmasonic p 30 H, Elma Schmidbauer GmbH, Baden-Württemberg, Germany) for 10 min, and vortexed for 30 s. Serial decimal dilutions (saline solution) were plated on CHROMagar Candida (Becton, Dickinson and Company, Difco, Franklin Lakes, NJ, USA) and brain heart infusion agar (Difco) supplemented with amphotericin B (Sigma-Aldrich) at 7 µg mL^−1^ to count the colonies of *C. albicans* and *S. mutans*, respectively. After 24 and 48 h of incubation, the number of viable cells was quantified, expressed in log_10_, and standardized per unit area (Log_10_ CFU cm^−^^2^) of HA discs.

#### 4.5.4. Biofilm pH Assessment

The biofilms treated with the E, TF2, ETF2 formulations, and CM were transferred to new sterile Falcon tubes and centrifuged for 5 min at 8000 rpm (Combi-514, Hanil Scientific Inc., Gyeonggi-do, Korea). The supernatant was filtered through a 0.22-µm membrane filter (Corning Inc.) and the biofilm pH was subsequently measured (pH electrode, 2A09E, Analyser).

### 4.6. Measurement of Inflammatory Cytokines TNF-α and IL-6

BMDMs were obtained as previously described [94,95], with some modifications. Briefly, bone marrow suspensions from femurs of 6-week-old C57BL/6 mice were grown in Roswell Park Memorial Institute 1640 medium (RPMI, Sigma-Aldrich) supplemented with 30% (*v*/*v*) L929-cell conditioned medium, 20% (*v*/*v*) fetal bovine serum (Gibco, Carlsbad, CA, USA), and 1% (*v*/*v*) penicillin–streptomycin (Sigma-Aldrich) to obtain BMDMs after 7 days of culture.

BMDMs were conditioned in a 96-well bottom plate (Nunc, Thermo Fisher Scientific) (2 × 10^5^ cells/well) and stimulated with LPS from *E. coli* (Sigma-Aldrich) at a concentration of 500 mg mL^−1^. After 4 h, the cells were washed with 1x phosphate-buffered saline and treated with the formulations (N, E, TF2, ETF2, and CM) for 2 h. The supernatant was collected for cytokine measurement, and the remaining cells were subjected to a cell viability assay MTT.

To evaluate the toxic effects of the formulations (ETF2, TF2, E, and CM), the BMDMs were treated for 2 h. Cell viability was determined using a 3-(4,5-dimethylthiazol-2-yl)-2,5-diphenyltetrazolium bromide colorimetric assay (MTT, Sigma-Aldrich [96]. Absorbances at 570 nm were measured on a microenzyme-linked immunosorbent assay (ELISA) reader (EL800, Gen5 Data Analysis Software, Agilent Technologies, Santa Clara, CA, USA). Three different assays were performed in triplicate.

TNF-α and IL-6 levels in the supernatant from the macrophage culture were determined using mouse TNF-α Quantikine ELISA (R&D Systems, Minneapolis, MN, USA) and IL-6 ELISA Set (BD Biosciences, San Diego, CA, USA) following the manufacturer’s instructions.

### 4.7. Statistical Analysis

All assays were performed in triplicate on at least three independent days. SigmaPlot software (version 12.0; Systat Software Inc., San Jose, CA, USA) and GraphPad PRISM (version 5.0) were used for statistical analyses, with a confidence level of 95%. Further, %SH, ΔKHN, and F, Ca, and N concentration in the enamel were analyzed by one-way analysis of variance, followed by Student–Newman–Keuls test. Anti-biofilm and inflammatory mediators’ data were analyzed using one-way analysis of variance, followed by post hoc tests of Holm–Sidak and Tukey’s multiple comparisons, respectively.

## 5. Patent

The patent for the mouthwash formulations was granted by *Instituto Nacional da Propriedade Industrial* (INPI, Brasília, Brazil) on 19 December 2019, with the process number of BR 10 2019 027251 1.

## Figures and Tables

**Table 1:** Mean (standard deviation) of the concentration of phenolic compounds (mg g^−1^) in samples.

Samples	Total Phenols Expressed as Gallic Acid
Pomegranate peel extract	114.98 (3.55)
N	0.49 (0.06)
E	11.56 (0.01)
TF1	0.54 (0.05)
TF2	0.53 (0.07)
ETF1	11.48 (0.22)
ETF2	11.59 (0.55)

N: formulation without fluoride (F),sodium-trimetaphosphate (TMP) and pomegranate peel extract (PPE); E: formulation with 3% PPE; TF1: formulation with 0.2% TMP + 100 ppm F; TF2: formulation with 0.3% TMP + 225 ppm F; ETF1: formulation with 3% PPE + 0.2% TMP + 100 ppm F); ETF2: formulation with 3% PPE + 0.3% TMP + 225 ppm F.

**Table 2:** Mean and standard deviation of variables’ surface hardness loss (%SH), subsurface hardness integrated loss (ΔKHN), fluoride, calcium, and phosphorus analyzed according to mouthwash formulation treatments.

Formulation	%SH (KHN)	ΔKHN (KHN × µm)	Fluoride (µg/mm^3^)	Calcium (µg/mm^3^)	Phosphorus (µg/mm^3^)
W	−87.4 ^a^ (3.2)	7249.8 ^a^ (782.5)	0.5 ^a^ (0.2)	150.7 ^a^ (31.0)	158.6 ^a^ (28.4)
E	−84.9 ^a^ (6.1)	6546.7 ^a^ (696.6)	0.5 ^a^ (0.4)	156.4 ^a^ (15.9)	143.5 ^a^ (44.0)
F1	−73.4 ^b^ (4.5)	4978.5 ^b^ (691.8)	0.6 ^b^ (0.2)	194.0 ^b^ (47.6)	170.0 ^b^ (36.0)
F2	−65.0 ^c^ (5.0)	3810.1 ^c^ (842.2)	1.2 ^c^ (0.4)	269.4 ^c^ (51.2)	215.0 ^c^ (10.9)
TF1	−56.4 ^d^ (4.4)	4309.4 ^c^ (497.7)	0.7 ^b^ (0.2)	212.3 ^b^ (85.3)	212.4 ^c^ (79.9)
TF2	−55.0 ^d^ (4.1)	3597.6 ^c^ (652.8)	1.2 ^c^ (0.4)	188.2 ^b^ (28.4)	196.6 ^c^ (35.4)
ETF1	−52.0 ^d^ (7.5)	3870.6 ^c^ (900.1)	0.8 ^d^ (0.2)	218.1 ^b^ (65.6)	210.9 ^c^ (48.3)
ETF2	−34.5 ^e^ (4.4)	2564.1 ^d^ (597.7)	1.2 ^c^ (0.7)	297.3 ^d^ (54.3)	204.4 ^c^ (43.8)
CM	−67.7 ^c^ (6.7)	5292.7 ^b^ (756.3)	1.2 ^c^ (0.1)	176.6 ^b^ (46.8)	210.5 ^c^ (32.1)

KHN: Knoop hardness; H: deionized water; E: formulation with 3% pomegranate peel extract (PPE); F1: formulation with 100 ppm fluoride (F); F2: formulation with 225 ppm F; TF1: formulation with 0.2% sodium trimetaphosphate (TMP) + 100 ppm F; TF2: formulation with 0.3% TMP + 225 ppm F; ETF1: formulation with 3% PPE + 0.2% TMP + 100 ppm F; ETF2: formulation with 3% PPE + 0.3% TMP + 225 ppm F; CM: essential oil commercial mouthwash. Different superscript letters indicate significant differences among the treatments for each variable separately. (One-way ANOVA, followed by Student–Newman–Keuls test; *p* < 0.001).

**Table 3:** Percentage of reduction in Candida albicans and Streptococcus mutans dual-biofilms formed for 24 h and treated for 1 or 10 min.

Biofilm-24 h	% Biofilm Reduction			
	*Candida albicans*		*Streptococcus mutans*	
Groups/Treatment (min)	1	10	1	10
E	20.26	31.74	24.54	27.93
TF2	17.36	32.07	10.55	28.50
ETF2	26.21	36.02	12.91	36.09
CM (positive control)	42.12	97.86	53.29	56.46
NC (negative control)	-	-	-	-

E: formulation with 3% pomegranate peel extract; TF2: formulation with 0.3% sodium-trimetaphosphate + 225 ppm of fluoride; ETF2: formulation with 3% pomegranate peel extract + 0.3% sodium-trimetaphosphate + 225 ppm of fluoride; CM: essential oil commercial mouthwash.

**Table 4:** pH values of *Candida albicans* and *Streptococcus mutans* dual-biofilms formed for 24 h and treated for 1 or 10 min.

Biofilm-24 h	pH	
	*Candida albicans* + *Streptococcus mutans*	
Groups/Treatment (min)	1	10
E	5.28	5.28
TF2	5.10	5.09
ETF2	5.15	5.20
CM (positive control)	5.67	5.51
NC (negative control)	5.45	5.51

E: formulation with 3% pomegranate peel extract; TF2: formulation with 0.3% sodium-trimetaphosphate + 225 ppm of fluoride; ETF2: formulation with 3% pomegranate peel extract + 0.3% sodium-trimetaphosphate + 225 ppm of fluoride; CM: essential oil commercial mouthwash.

**Table 5:** Groups of mouthwash formulations designed according to their constituents (g).

Constituent	Mouthwash Formulation							
	N	E	F1	F2	TF1	TF2	ETF1	ETF2
Pomegranate Peel Extract	-	10.40	-	-	-	-	10.40	10.40
Stabilizers	0.50	0.50	0.50	0.50	0.50	0.50	0.50	0.50
Microbiological Conserver	0.10	0.10	0.10	0.10	0.10	0.10	0.10	0.10
Chelating	0.01	0.01	0.01	0.01	0.01	0.01	0.01	0.01
Sodium-Fluoride	-	-	0.02	0.05	0.02	0.05	0.02	0.05
Sodium-Trimetaphosphate	-	-	-	-	0.20	0.30	0.20	0.30
Sweetener I	7.50	7.50	7.50	7.50	7.50	7.50	7.50	7.50
Humectant	10.00	10.00	10.00	10.00	10.00	10.00	10.00	10.00
Purified water q.s.	100	100	100	100	100	100	100	100

N: formulation without fluoride (F); sodium-trimetaphosphate (TMP) and pomegranate peel extract (PPE); E: formulation with 3% PPE; F1: formulation with 100 ppm F; F2: formulation with 225 ppm F; TF1: formulation with 0.2% TMP + 100 ppm F; TF2: formulation with 0.3% TMP + 225 ppm F; ETF1: formulation with 3% PPE + 0.2% TMP + 100 ppm F; ETF2: formulation with 3% PPE + 0.3% TMP + 225 ppm F.

## Data Availability

Not applicable.

## Supplementary Materials

The following supporting information can be downloaded at: https://www.mdpi.com/article/10.3390/antibiotics11111477/s1, Figure S1: Scanning electron microscopy images analysis of enamel after treatment with ETF2 x5000 (A), ×10,000 (B), and ×20,000 (C); Table S1: Means (SD) of *C. albicans* and *S. mutans* cells at different times (T0-T10) after being treated with pomegranate peel extract diluted in propylene glycol at different concentrations (1, 3, and 6%).
