# Supplementary material for: Pomegranate Extract Potentiates the Anti-Demineralizing, Anti-Biofilm, and Anti-Inflammatory Actions of Non-Alcoholic Mouthwash When Associated with Sodium-Fluoride Trimetaphosphate
Source: Antibiotics (Basel). 2022 Oct 25;11(11):1477. doi: 10.3390/antibiotics11111477 (PMC9686636; doi:10.3390/antibiotics11111477)
Supplement: Supplementary file 1 [file antibiotics-11-01477-s001.zip › antibiotics-1924221-supplementary.pdf]

Supplementary Materials

Ingredients of essentials oil anticaries alcohol-free commercial mouthwash

Purified water, sorbitol, propylene glycol, poloxamer 407, sodium lauryl sulfate, benzoic acid, aroma (benzyl alcohol, d-Limonene), eucalyptol, methyl salicylate, thymol, sodium saccharina, sodium fluoride (220 ppm), sodium benzoate, sucralose, menthol, CI 47005/quinoline yellow, CI 42053/fast green.

Microbiological test for selecting pomegranate peel extract concentration in mouthwash formulations

Pomegranate peel extract (PPE) at concentrations of 1%, 3%, and 6% were diluted in propylene glycol. To determine the concentration of PPE in mouthwash formulations with the most antimicrobial effect, cell death was induced according to de Castro et al. [81] against microorganisms (*C. albicans* ATCC 10231 and *S. mutans* ATCC 35668). PPE was in contact with the microorganisms for 0.5, 1, 2, 3, 4, 5, and 10 min. Next, the minimum inhibitory concentration (MIC) of PPE was determined using the broth microdilution method against these strains following the norms established by the Clinical and Laboratory Standards Institute (Documents M27-A2 and M07-A9), with some modifications. The assays were performed in triplicate on three different occasions, as described in Suppementary Table S1.

Table S1. Means (SD) of *C. albicans* and *S. mutans* cells at different times (T0-T10) after being treated with pomegranate peel extract diluted in propylene glycol at different concentrations (1, 3 and 6%).

	**Log_{10} CFU cm^{-2} *Candida albicans***							
	T0	T0.5	T1	T2	T3	T4	T5	T10
**PPE 1%**	6.40 (0.09)	5.17 (0.15)	6.10 (0.72)	5.49 (0.07)	5.31 (0.03)	5.46 (0.09)	5.04 (0.22)	5.60 (0.14)
**PPE 3%**	5.77 (0.04)	5.33 (0.08)	5.33 (0.11)	5.68 (0.03)	5.37 (0.37)	5.61 (0.13)	4.68 (0.81)	5.78 (0.04)
**PPE 6%**	5,87 (0.09)	5.33 (0.04)	5.46 (0.24)	5.78 (0.05)	5.28 (0.19)	5,83 (0.06)	5.42 (0.20)	5.92 (0.24)

	**Log_{10} CFU cm^{-2} *Streptococcus mutans***							
	T0	T0.5	T1	T2	T3	T4	T5	T10
**PPE 1%**	8.56 (0.06)	8.35 (0.24)	8.44 (0.25)	8.50 (0.09)	8.52 (0.12)	8.42 (0.07)	8.53 (0.08)	8.59 (0.01)
**PPE 3%**	8.54 (0.11)	8.53 (0.08)	8.43 (0.05)	8.27 (0.66)	5.49 (1.80)	8.02 (0.02)	8.15 (0.14)	7.91 (0.10)
**PPE 6%**	8.41 (0.16)	8.31 (0.15)	8.20 (0.03)	7.69 (0.09)	8.03 (0.11)	7.84 (0.01)	7.93 (0.05)	7.48 0.03)

The PPE was effective against both microorganisms, with MIC values of 0.97 and 1.95 mg/mL^{-1} respectively for *C. albicans* and *S. mutans*. Thus, as there were no differences between the concentrations of 1, 3, and 6%, considering the treatment time of 1 minute, the concentration of 3% PPE was selected for the development of mouthwash formulations.

Analysis of the enamel surface using scanning electron microscopy

Enamel blocks (n = 1) were analyzed by scanning electron microscopy at 5000, 10000 and 20000 x magnifications to obtain images of the enamel surface after being subjected to pH cycling and treatment with the ETF2 formulation. The block was dehydrated in ethanol (50, 60, 70, 80, and 100%) and gold-plated (Quorum Q150 TE, Quorum Technologies, Laughton, United Kingdom). The instrument used was an MEV-PHILIPS (XL-30 FEG, Philips, Amsterdam, Netherlands) [84].

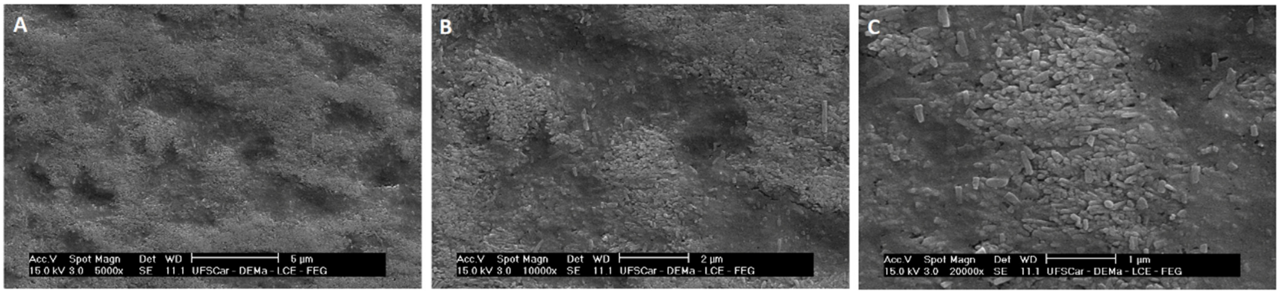

Figure S1. Scanning electron microscopy images analysis of enamel after treatment with ETF2 x5000 (A), x10000 (B) and x20000 (C).

Artificial saliva

Artificial saliva used in the biofilm assays was based on the protocol of Lamfon et al. [92] and was comprissed of:1 L of deionized water, 2 g yeast extract (Sigma-Aldrich), 5 g bacteriological peptone (Sigma-Aldrich), 2 g glucose (Sigma-Aldrich), 1 g mucin from porcin stomach (Sigma-Aldrich), 0.35 g NaCl (Sigma-Aldrich), 0.2 g $CaCl_2$ (Sigma-Aldrich), and 0.2 g KCl (Sigma-Aldrich). The final pH was adjusted to 6.8 using NaOH (Sigma-Aldrich).

Preparation of hydroxyapatite discs

Hydroxyapatite (HA) discs were prepared and characterized as described by Arias et al. [93]. HA powder (0.650 g; Sigma-Aldrich) was added to a stainless-steel die (SKAY, São José do Rio Preto, Brazil) and placed in a hydraulic press, where it was subjected to a load of 25 tons. The discs were 13 mm in diameter and 3 mm thick. Before performing the biofilm assays, they were sterilized using ethylene oxide (Oximed–*Tecnologia em esterelização* – São José do Rio Preto, SP, Brazil).
